# Fast and Accurate Prediction of Tautomer Ratios in Aqueous Solution via a Siamese Neural Network

**DOI:** 10.1021/acs.jctc.5c00041

**Published:** 2025-03-17

**Authors:** Xiaolin Pan, Xudong Zhang, Song Xia, Yingkai Zhang

**Affiliations:** † Department of Chemistry, New York University, New York, New York 10003, United States; ‡ Simons Center for Computational Physical Chemistry at New York University, New York, New York 10003, United States; § NYU-ECNU Center for Computational Chemistry at NYU Shanghai, Shanghai 200062, China

## Abstract

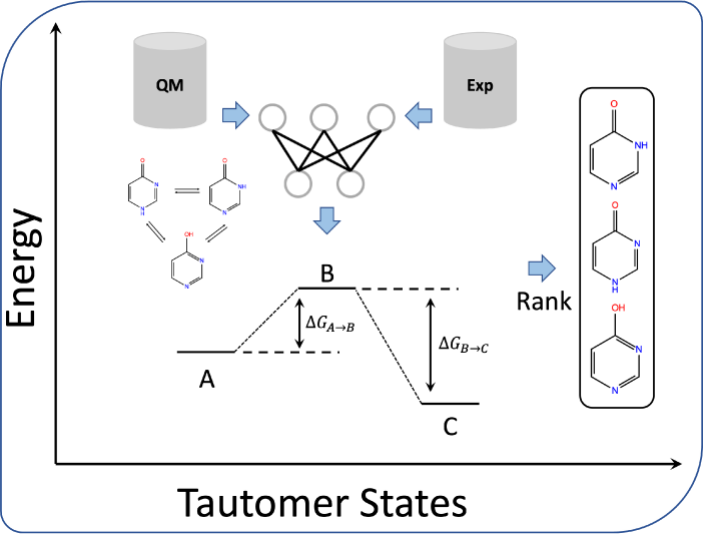

Tautomerization plays a critical role in chemical and biological processes, influencing molecular stability, reactivity, biological activity, and ADME-Tox properties. Many drug-like molecules exist in multiple tautomeric states in aqueous solution, complicating the study of protein–ligand interactions. Rapid and accurate prediction of tautomer ratios and identification of predominant species are therefore crucial in computational drug discovery. In this study, we introduce sPhysNet-Taut, a deep learning model fine-tuned on experimental data using a Siamese neural network architecture. This model directly predicts tautomer ratios in aqueous solution based on MMFF94-optimized molecular geometries. On experimental test sets, sPhysNet-Taut achieves state-of-the-art performance with root-mean-square error (RMSE) of 1.9 kcal/mol on the 100-tautomers set and 1.0 kcal/mol on the SAMPL2 challenge, outperforming all other methods. It also provides superior ranking power for tautomer pairs on multiple test sets. Our results demonstrate that fine-tuning on experimental data significantly enhances model performance compared to training from scratch. This work not only offers a valuable deep learning model for predicting tautomer ratios but also presents a protocol for modeling pairwise data. To promote usability, we have developed an accessible tool that predicts stable tautomeric states in aqueous solution by enumerating all possible tautomeric states and ranking them using our model. The source code and web server are freely accessible at https://github.com/xiaolinpan/sPhysNet-Taut and https://yzhang.hpc.nyu.edu/tautomer.

## Introduction

Tautomeric equilibrium plays a crucial role in various chemical and biological processes, affecting molecular stability, reactivity, and biological activity. In drug discovery, studying tautomeric equilibrium is essential because many drug-like compounds exhibit heterocyclic structures with potential tautomeric transformations.^1,2^ Notably, approximately 26% of approved drugs exist in different tautomeric states.^3^ Tautomeric interconversions mainly involve three types: prototropic tautomerism, ring–chain tautomerism, and valence tautomerism. Prototropic tautomerism is the most common type in drug molecules, involving bond reformation and proton transfer. This transformation interchanges pharmacophore types with hydrogen bond donors becoming acceptors and vice versa, which alters the interaction between proteins and ligands.^1,3^ Some studies have discussed the effects of tautomerization on structure-based and ligand-based screening methods, where high-energy tautomer states may form different interactions that lead to an increase in false positives and unnecessary computational costs.^4−9^ Correctly assigning tautomer states in protein–ligand complexes is also crucial for molecular dynamic simulations and protein–ligand binding free energy calculations.^10,11^ Several experimental techniques are available to determine the tautomer ratio in various solutions, including NMR, UV–visible spectroscopy, IR, and fluorescence spectroscopy.^12−14^ However, the small free energy differences between tautomeric states and their rapid interconversion make experimental measurements challenging. Therefore, rapid and accurate prediction of tautomer ratios and favorable tautomeric states in aqueous solution is essential for computational drug discovery.

Traditional computational methods are generally based on empirical rules or quantum mechanical (QM) calculations. Empirical rules-based methods^4,15−19^ rely on rules derived from experimental and computed data to determine the tautomeric preference, considering factors like the number of aromatic rings and double bonds. However, empirical-based scoring methods only rank tautomeric states without providing energy information. Quantum mechanical calculations-based methods, which combine QM method and an implicit solvent model in a thermodynamic cycle, can accurately calculate energy differences between tautomer states. While these methods offer remarkable performance, their substantial computational requirements limit high-throughput applications. The tautomer ratios can be converted into free energies by ΔG = -RTln *K*, allowing for accurate calculation of it through free energy calculation methods. In the SAMPL2 blind challenge, designed to evaluate computational methods for predicting hydration free energies and tautomer ratios in aqueous solution, the top four submissions used quantum mechanical methods with implicit solvent models, and they achieved root-mean-square error (RMSE) deviations ranging from 1.9 to 3.4 kcal/mol.^20−23^ Additionally, Wieder et al.^24^ achieved comparable results with an RMSE of 2.2 kcal/mol in a retrospective study using B3LYP/aug-cc-pVTZ//B3LYP/6–31G(d)/SMD.

Although quantum mechanical based methods have achieved some success in predicting tautomer ratios, their accuracy is limited, and they require significant computational resources. Recently, deep learning strategies have made progress in predicting electronic energies, solvation energies, atomic forces, and various molecular properties. One approach is to develop deep potentials to improve the accuracy of molecular simulations by learning density functional theory (DFT) calculated energies, forces, and partial charges.^25−36^ This type of method optimizes molecular geometries iteratively by itself and then calculates the equilibrium electronic energy. It also requires significant computational time for processing large chemical library, although it is faster than DFT methods. Another approach is to learn the electronic energy and solvation energy using force field-optimized geometries.^37−39^ This method saves a lot of computational resources and time by avoiding the need to obtain high-quality geometries while still achieving good accuracy. Advances in artificial intelligence offer new avenues for developing methods that can predict tautomer ratios in aqueous solutions quickly and accurately. Wieder et al.^24^ also utilized experimental data to fine-tune the ANI-1ccx deep potential model for predicting tautomer ratios in aqueous solution, including solvent effects by employing a relative alchemical free energy calculation protocol. Their optimized ANI-1ccx model achieved an RMSE of 2.8 [2.2, 3.2] kcal/mol with the alchemical free energy calculation, which is better than the native ANI-1ccx model’s performance with an RMSE of 6.7 [5.7, 7.7] kcal/mol. Ji et al.^40^ developed a tool for predicting favorable tautomeric states, which includes a deep learning-based scoring method for tautomer ranking. This scoring method combines the ANI-2x deep potential with a deep learning-based solvation model trained on DFT-calculated data, which achieved a similar performance to wB97*X*/6–31G*//M062*X*/6–31G*/SMD on the experimental data set, with an RMSE of 3.15 kcal/mol. All these methods are somewhat time-consuming or have limited performance. The method developed by Wieder et al. needs to run MD and alchemical free energy calculations; the method developed by Ji et al. requires optimizing molecular geometries using the ANI-2x deep potential model and its performance cannot surpass that of the DFT methods it was trained on. To our best knowledge, no deep learning approaches have developed to directly predict experimental tautomer ratios based on the input of two tautomer structures. Meanwhile, due to the limited size of experimental data, to train a robust and accurate deep learning model from scratch can be challenging.

**Figure 1:**
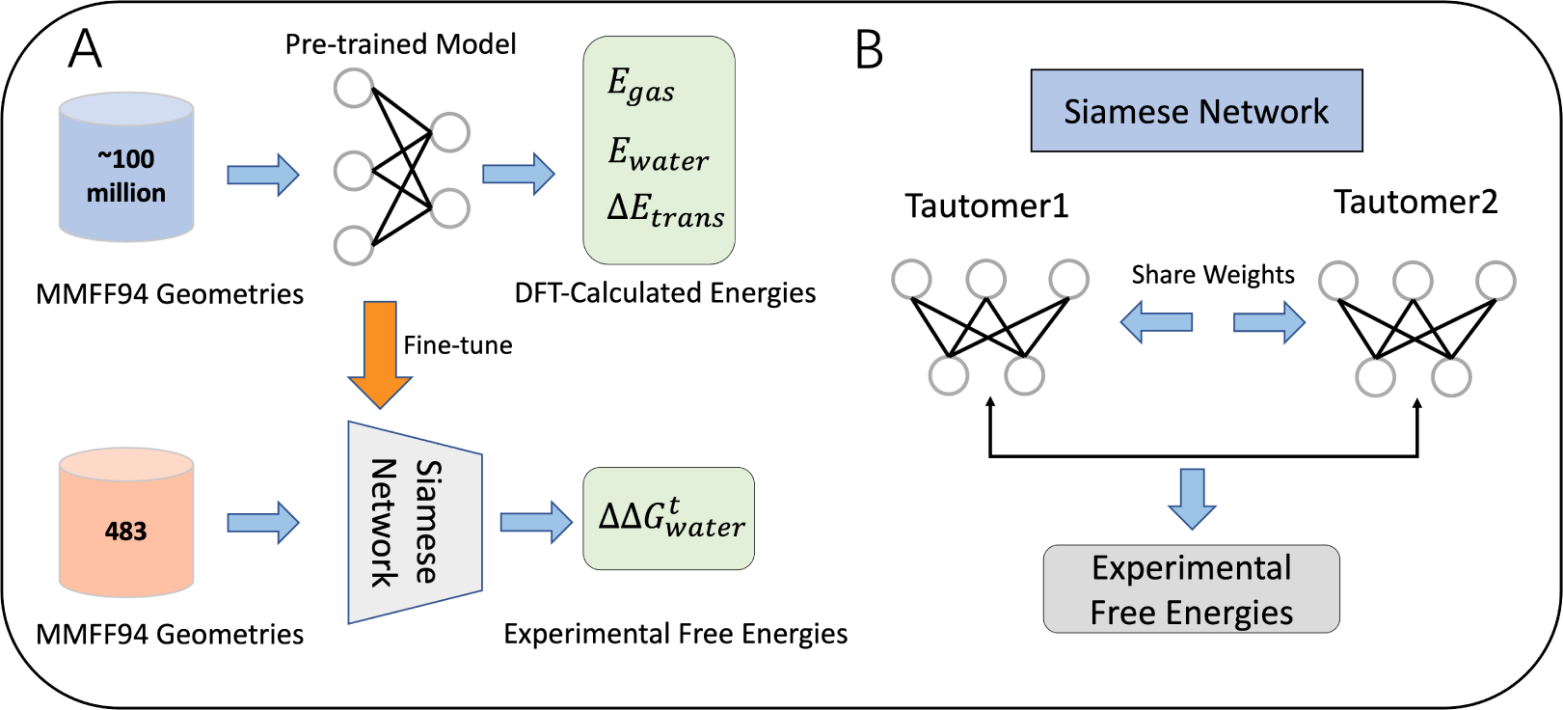
A strategy to develop a deep learning model for predicting tautomer ratios in aqueous solution with limited experimental data, based only on the structures of two tautomeric states. (A) The pretrained model was first trained on data from B3LYP/6–31G*/SMD calculations and subsequently fine-tuned with experimental data. This approach enables the prediction of relative free energies between tautomer pairs using a Siamese neural network. (B) The Siamese neural network architecture. The inputs are two tautomeric states with 3D geometries, and the model predicts their free energy difference. Basic models are applied to each input, sharing their weights.

In this study, we introduce sPhysNet-Taut, a model fine-tuned from the pretrained neural network using experimental data. This model takes MMFF94-optimized conformations as input and directly predicts the relative energy between tautomer pairs. We constructed the Frag20-Taut database using DFT calculations, which includes electronic energy data for both gas and aqueous phases. This data set contains approximately one million well-selected molecules. Initially, we trained the sPhysNet-MT model on the Frag20-Taut data set to predict calculated energies. Subsequently, we designed a Siamese neural network^14,41^ based on sPhysNet-MT and fine-tuned it using experimental data collected from Tautobase^42^ (as shown in Figure 1). After training and fine-tuning on the Frag20-Taut and experimental data sets using 5-fold cross-validation, our model significantly outperforms other methods, achieving an RMSE of 1.9 kcal/mol on the 100-tautomers set and 1.0 kcal/mol on the SAMPL2 challenge. On an external test set, our model achieved a 77% success rate in ranking the favorable tautomer as the preferred species. Additionally, we developed a user-friendly web server and command line tool for tautomer enumeration and ranking in aqueous solution based on the sPhysNet-Taut model. The web server is available at https://yzhang.hpc.nyu.edu/tautomer, and the source code is accessible at https://github.com/xiaolinpan/sPhysNet-Taut.

## Material and Methods

### Data Set

#### Data Set for Calculated Energetics: Frag20-Taut

**Figure 2:**
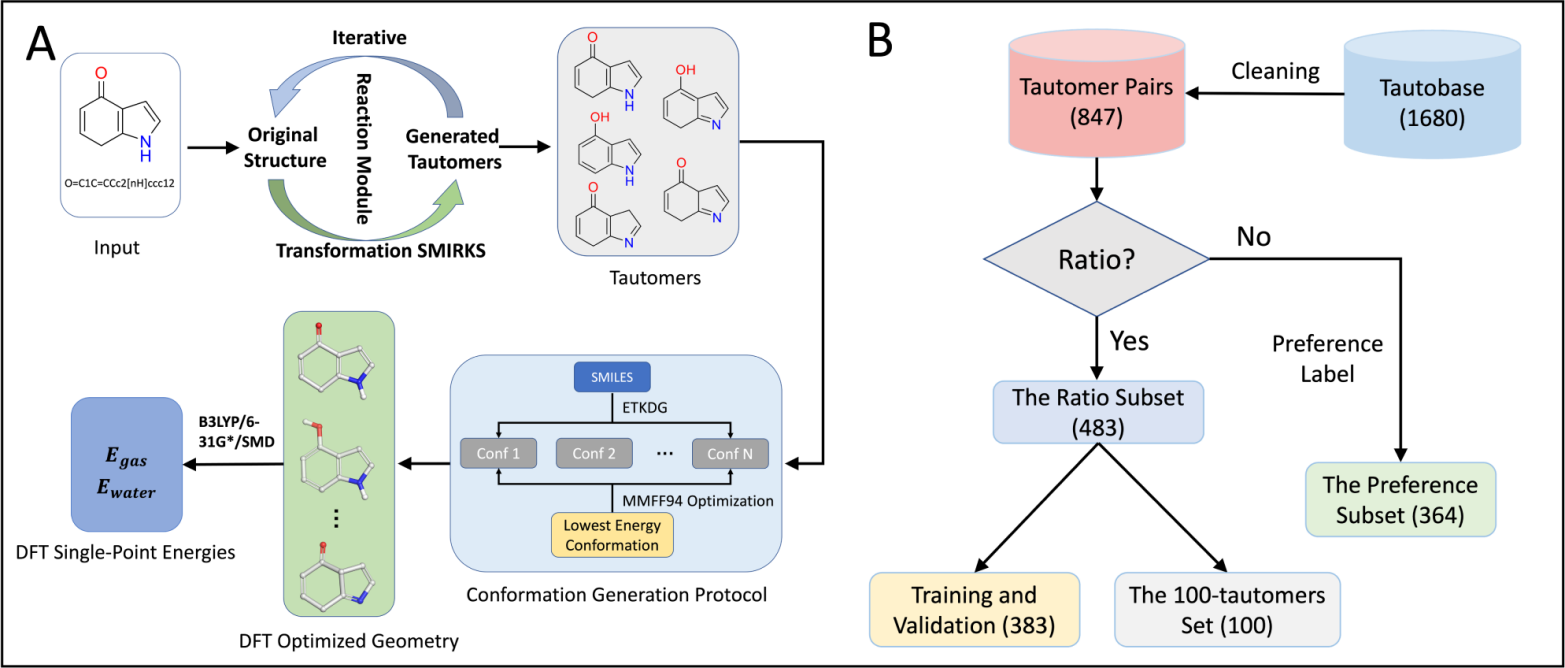
(A) Workflow for preparing Frag20-Taut data set. This process includes four steps: Enumerating all possible tautomeric states using transformation rules, optimizing the conformation of each tautomer using the MMFF94 force field and selecting the lowest-energy conformation, performing geometry optimization using B3LYP/6–31G*/SMD, and calculating single-point energies. (B) The workflow used to compile experimental data from the Tautobase database. Two data sets were extracted: the ratio subset with experimentally measured log K values, and the preference subset with experimental preferred states.

To train sPhysNet-MT for accurately predicting the relative energies between different tautomeric states in aqueous solution, we constructed the Frag20-Taut data set comprising a large set of generated tautomer pairs. In our previous work, we developed the Frag20 data set to model molecular electronic energies and transfer energies. The molecules within Frag20 data set were meticulously selected using a well-designed protocol to ensure sufficient diversity while limiting the maximum heavy atom count to 20, as detailed in our earlier publications.^37,39^ We adapted the SMIRKS strings of the 54 prototropic tautomeric transformation rules summarized by Dhaked et al. for compatibility with the RDKit reaction module.^40,43^ These transformation rules were applied to generate tautomer pairs for molecules within the Frag20 data set. If the 54 prototropic tautomeric rules did not yield tautomeric states, we employed the tautomer generation module in RDKit. We randomly selected 62,688 molecules with potential tautomeric transformation from Frag20, generating a total of 313,630 tautomeric molecules. We retained only the molecules whose SMILES strings did not change after DFT optimization, resulting in a total of 250,822 molecules. The Frag20-Taut combines Frag20 and the generated tautomeric molecules, containing a total of 929,738 molecules with calculated energies. Figure 2 illustrates the preparation workflow for Frag20-Taut. The first step involves generating all possible tautomeric states for each molecule and discarding those without alternative tautomeric states. The second step is to generate conformations for each tautomer structure using the ETKDG method^44,45^ and optimize them with the MMFF94 force field.^46−50^ For each tautomer, we generated 300 initial conformations and optimized them using MMFF94, retaining the lowest-energy optimized conformation as the input geometry for DFT calculations. The third step involves optimizing the geometric structures and calculating the electronic energies E_gas_ and E_water_ using the DFT method at the B3LYP/6–31G* level with the universal solvation model (SMD) in Gaussian 16 software.^51^ The transfer energies are determined by calculating the difference between the single-point electronic energies in the gas phase and the water phase, defined as follows: ΔE_transfer_ = E_water_ – E_gas_.

#### Experimental Tautomer Data Set: Tautobase

Wahl et al. have published an open-source tautomer database, Tautobase, containing experimentally measured and estimated tautomer ratios for 1680 entries in various solvents, primarily water.^42^ In this study, our goal is to develop a model for predicting tautomer ratios to accelerate drug discovery, focusing on water as the solvent since the tautomeric state within high abundance preferentially binds to receptor. To obtain a clean experimental data set for fine-tuning our model, we filtered records in Tautobase using the following criteria: (1) only records measured in water were retained; (2) the record must have a logK value; (3) all molecules must be neutral; (4) the elements in the molecule must be limited to C, H, O, N, S, P, F, Cl, and Br; (5) the tautomerism must be a prototropic transformation. After applying these rules and excluding molecules from the SAMPL2 challenge, we obtained 483 records and named it as the ratio subset. Additional records without logK values in water were used as an external test set to evaluate the ranking power of our model, defined by its ability to prioritize the most favorable tautomer as the top-ranked species. The external test set contains a total of 364 records and named it as the preference subset. We then use the same protocol as in the Frag20-Taut preparation to generate the lowest-energy MMFF94-optimized conformation for each tautomer from the SMILES string. The logK values were converted to free energy by ΔG = = RTln *K*.

### Deep Learning Models

#### Siamese Neural Network

**Figure 3:**
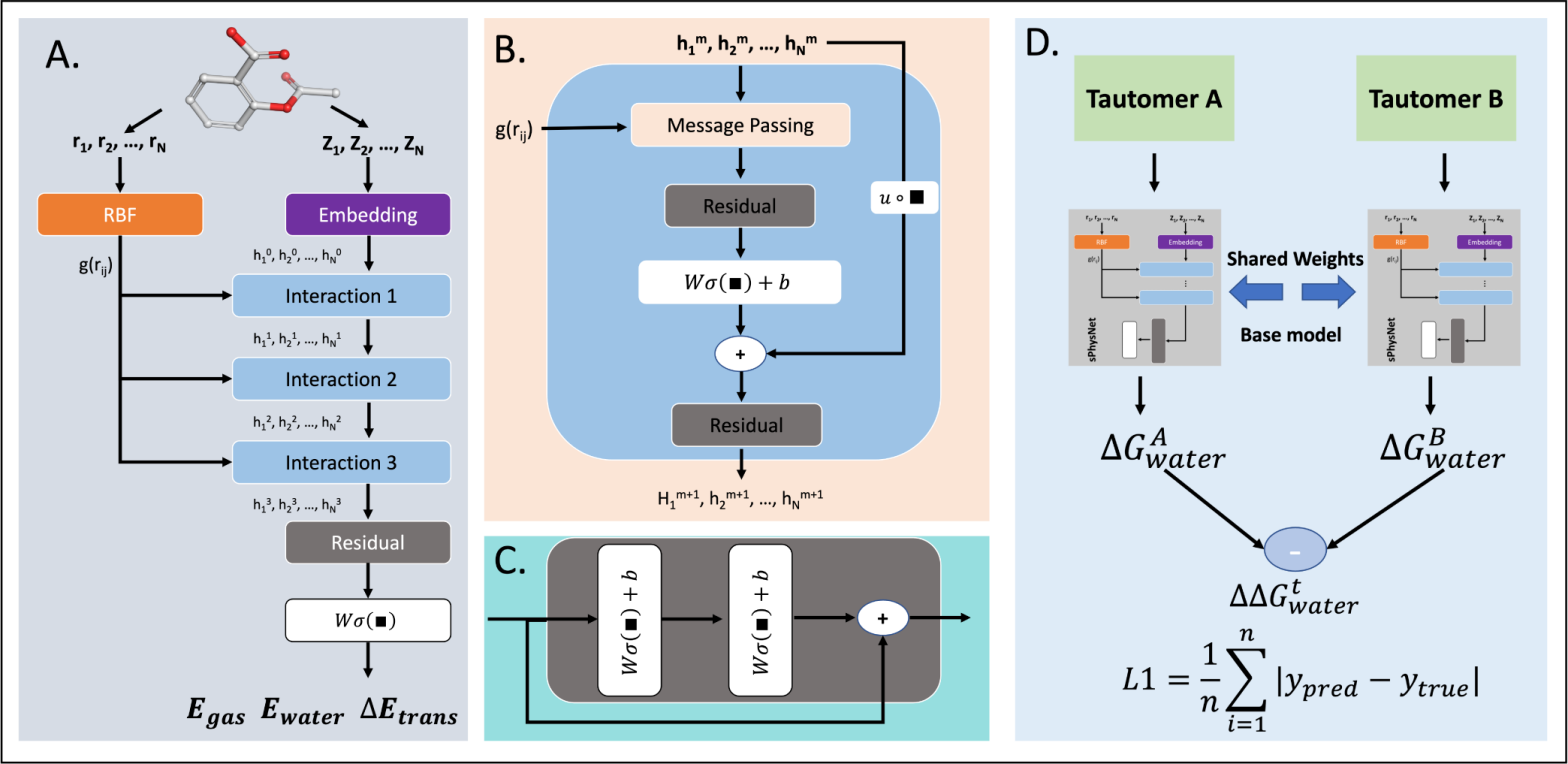
Overview of the sPhysNet-Taut model based on sPhysNet^37,39^ and PhysNet.^34^ (A) The architecture of the sPhyNet-MT model. It consists of a radial basis function (RBF) layer, an embedding layer, interaction layers, and residual layers. The RBF layer encodes distances between atom pairs into vectors, while the embedding layer encodes element types into vectors. This information is then processed through the interaction layer and residual layer to predict the final targets. (B) The interaction layer, which includes message passing, residual layers, and gate layers. (C) The residual layer, used within the interaction layer and before the model output, helps refine the predictions. (D) The architecture of the Siamese neural network. The core of the Siamese neural network is the sPhysNet-MT model with shared weights across both branches. The output is calculated as the difference between the predicted energies in water for the two mirrored models.

Siamese neural networks^14,41^ are commonly used to process two inputs and determine the distance between them, such as in object tracking and matching tasks. The goal of model optimization is to minimize the distance between the predicted vectors for inputs of the same category and maximize it for those of different categories, as shown in eq 1, where *f*(*x*) represents any differentiable function. These neural networks are also suitable for modeling differences between two relative states to predict pairwise properties, such as protein–ligand relative binding affinities^52^ and molecular property predictions.^53^ In this work, we designed a Siamese neural network based on the pretrained sPhysNet-MT model and fine-tuned its parameters using experimental data. The model architecture is detailed in Figure 3D. Two input tautomeric states are processed by the shared base model to predict their electronic energies in water. The relative energies, , are calculated as the difference between the two predicted energies, as shown in eq 2. These two base models share their weights, ensuring consistent processing of both inputs.
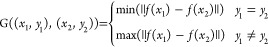
1
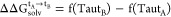
2

#### sPhysNet-MT

To accurately predict the DFT calculated electronic energies in both gas and aqueous phases, we developed a multitask deep learning model called sPhysNet-MT, using MMFF94-optimized conformations as input structures. sPhysNet-MT is a modification of PhysNet,^34^ featuring fewer parameters, faster computation speed, and is implemented using PyTorch. Figure 3A–C illustrate the architecture of sPhyNet-MT, which includes an RBF layer, an element-type embedding layer, interaction modules, and residual modules. Initially, the N atoms in a molecule go through an embedding layer and are embedded into vectors  (node embeddings). The distances between each atom pair are expanded with RBFs into vectors {g(rij) | i, j ∈ {1,2,···, N}, rij <10Å} (edge embeddings). The node embeddings are then updated by three interaction modules with message passing, residual layers, and gate layers. The node embeddings of the final layer go through the output layer to predict targeted properties. For each input molecule, sPhysNet-MT provides three predicted energies by summing the predicted atomic energies: electronic energies both in the gas phase and the aqueous phase, and transfer energies between these phases. The details of each module are described in Text S1.

### Model Training

#### Pretraining on Frag20-Taut

Since the experimental data on the relative free energy between tautomer pairs is extremely limited, it is difficult to train a robust and accurate deep learning model from scratch. Therefore, as described earlier, we aim to pretrain our model based on DFT-calculated data to obtain good initial model parameters, ensuring fast convergence and improved accuracy when fine-tuning the model based on experimental data, while also integrating the robustness of the DFT method. The Frag20-Taut data set comprises three target energies: E_gas_, E_water_, and ΔE_trans_. To train the multitask model on these properties, we employ a loss function that optimizes the model weights by summing the L1 losses for each of the three energies. The loss function is defined as follows:
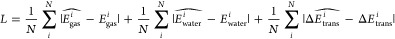
3

We randomly split the Frag20-Taut data set into a training set, validation set, and test set, containing 893,954, 17,942, and 17,844 samples, respectively. Training a model based on DFT-optimized geometries is often impractical for high-throughput energy calculations; therefore, we used MMFF94-optimized geometries as input structures for the pretrained model. For comparison, we also trained a model using DFT-optimized geometries to assess the performance differences attributable to input geometries. sPhysNet-MT was implemented using PyTorch, with the message-passing module and data loader module based on PyTorch Geometric (PyG).^54^ We used the EmaAmsGrad optimizer with a learning rate of 0.001, a β parameter set from 0.0 to 0.99, epsilon at 1e-8, and no weight decay. During training, we saved the model weights that achieved the best performance (RMSE) on the validation set over 500 epochs, utilizing an NVIDIA A100 80GB GPU.

#### Fine-Tuning on Experimental Data

Our objective with the experimental data set was to fine-tune the predictions of tautomer pairs in aqueous solution using MMFF94-optimized geometries within our designed Siamese neural network. We utilized 5-fold cross-validation to train and validate the neural network. The experimental data set was split into a training set and a validation set in an 8:2 ratio, resulting in 306 tautomer pairs for training and 77 for validation. The test set comprised a fixed set of 100 tautomer pairs, referred to as the “100-tautomers set”. For gas-phase energies, we used DFT-calculated values as learning targets. We employed the EmaAmsGrad optimizer in our fine-tuning process over 2,000 epochs with a learning rate of 0.0001, saving the model weights that exhibited the best performance on the validation set. An L1 loss function was used to optimize the model weights, with α and β set to 0.8 and 0.2, respectively. The loss function is defined as follows:
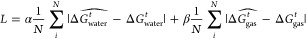
4

### Workflow for Predicting Favorable Tautomers

In this work, we have developed a rapid workflow to predict favorable tautomeric states in aqueous solution. Initially, we enumerate all possible tautomeric states using transformation rules in the RDKit reaction module, covering 54 types of prototropic tautomerism summarized by Dhaked et al.^43^ For each input molecule, we apply these transformation rules iteratively up to five times using a cycle generation protocol. We generate molecular conformations using ETKDG^44,45^ and optimize each one using MMFF94^46−50^ within RDKit package, retaining the lowest-energy conformation as the input for our energy prediction model. Next, we predict the relative energy of each tautomer pair and rank the tautomer states based on their predicted relative energies in water. Finally, we identify all low-energy tautomeric states based on an energy cutoff (default is 2.76 kcal/mol, corresponding to 1% tautomer ratio). To evaluate the runtime of our workflow, we selected 483 molecules from the ratio subset as a molecular pool to generate tautomeric states and conformations, which we then ranked using our model. All calculations were running on a single CPU core. Our workflow can process an average of 14.4 molecules per minute. In comparison, MolTaut can only process an average of 1.8 molecules per minute, and the DFT method can process only 0.005 molecules per minute. Our workflow is about 2880 times faster than the DFT method and about 8 times faster than MolTaut.

## Results and Discussion

### Performance in Predicting Relative Free Energies between Tautomer Pairs

In a previous study focused on favorable tautomer prediction, Ji et al.^40^ developed a scoring method called MolTaut for tautomer ranking, which combines the ANI-2x deep potential with a deep learning-based solvation model (MolSolv) trained on DFT-calculated data. However, the performance of MolTaut was limited by the inherent constraints of DFT methods. To enhance accuracy, we aimed to integrate both calculated and experimental data. As previously mentioned, we fine-tuned a pretrained model using experimental data within a Siamese neural network, resulting in the sPhysNet-Taut model. A detailed description of the performance for the pretrained model is provided in the Text S2.

**Figure 4:**
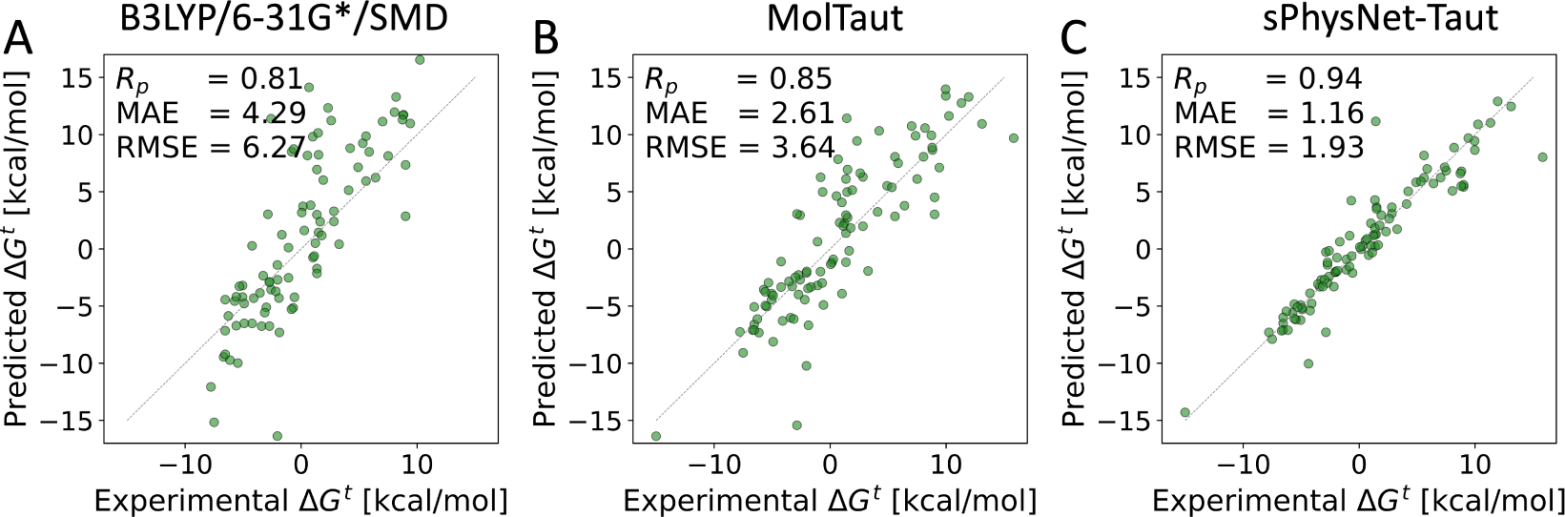
Comparative performance of three methods for predicting the experimental relative free energy between tautomer pairs in aqueous solution: (A) B3LYP/6–31G*/SMD: Uses the B3LYP functional with the 6–31G* basis set the SMD solvation model. (B) MolTaut: Uses ANI-2x for predicting electronic energy in the gas phase and the MolSolv model for solvation energy prediction, (C) sPhysNet-Taut: Fine-tuned on experimental data using MMFF94-optimized geometries.

To comparatively evaluate the performance of our model, we compared the performance of B3LYP/6–31G*/SMD, MolTaut, and sPhysNet-Taut model based on MMFF94 optimized geometries. As illustrated in Figure 4, sPhysNet-Taut predicts the relative free energies on the **100-tautomers set** with a MAE of 1.16 kcal/mol and an RMSE of 1.93 kcal/mol. This performance significantly outperforms both the MolTaut scoring method (MAE = 2.61 kcal/mol, RMSE = 3.64 kcal/mol) and B3LYP/6–31G*/SMD (MAE = 4.29 kcal/mol, RMSE = 6.27 kcal/mol), achieving state-of-the-art results. Additionally, we analyzed the distribution of prediction errors across different ranges of heavy atom counts in the test set (Figure S1). For molecules with fewer than 15 heavy atoms, we observed that the prediction error increased with molecular size. In contrast, for molecules with more than 15 heavy atoms, the prediction error did not further increase, which may be attributed to data set imbalance. Overall, these findings suggest that molecular size plays a significant role in influencing the performance of the model.

SAMPL2,^55^ a blind computational challenge, provides a list of tautomer pairs with experimentally measured ratio values, making it an excellent data set for evaluating various models. This data set is split into two subsets: an obscure set containing 8 tautomer pairs and an explanatory set with 12. We selected tautomer pairs that contain only neutral molecules with reliable error estimations. These molecules were excluded from our training set to ensure unbiased evaluation. We assessed the performance of our model against four methods that excelled in the SAMPL2 challenge (refs.^20−23^), one method distinguished for its performance in a retrospective study (ref,^24^ and the previously proposed AI-based method, MolTaut. Further details on these methods are available inText S3. According to the results shown in Tables 1 and S2, our sPhysNet-Taut model achieved a total RMSE of 1.0 kcal/mol with MMFF94-optimized geometries, greatly outperforming other quantum mechanics-based methods and MolTaut. Notably, whereas most methods showed less robust performance on the explanatory set compared to the obscure set (except for ref,^20^ our method demonstrated the opposite trend, excelling on the explanatory data set with an RMSE of 0.8 kcal/mol using MMFF94-optimized geometries. Furthermore, as illustrated in Figure S2, most tautomeric transformations in the SAMPL2 test set involve 1,3 aromatic heteroatom H-shifts, which are common and crucial in drug-like heterocyclic compounds. This result suggests that sPhysNet-Taut is capable of accurately predicting tautomer ratios for this type of tautomerization.

**Table 1:** Performance of This Work, Wieder et al.’s Method, and Several Submissions of SAMPL2 Challenge

	RMSE (kcal/mol)		
name	the obscure set	the explanatory set	ref
sPhysNet-Taut	1.3	0.8	this work
MolTaut^40^	1.3	3.9	(40)
Wieder et al.^24^	1.3	2.5	(24)
Klamt et al.^21^	1.4	3.6	
Ribeiro et al.^22^	1.5	2.9	
Kast et al.^20^	2.8	0.8	
Soteras et al.^23^	1.3	3.8	

**Figure 5:**
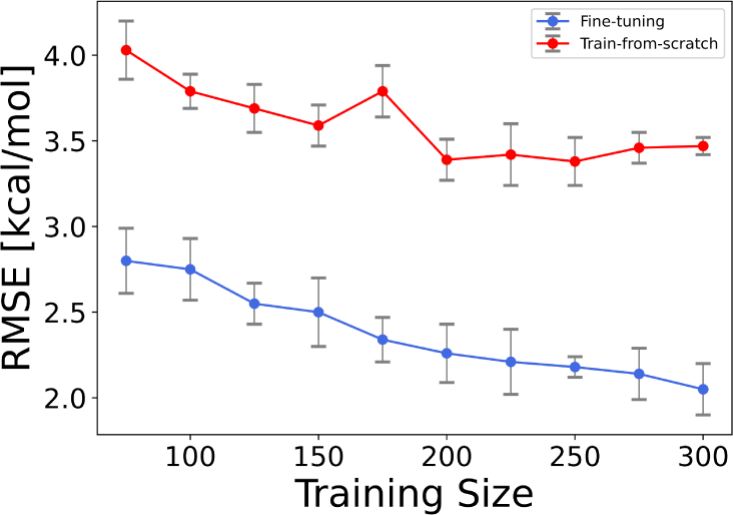
Performance of sPhysNet-Taut on the 100-tautomers test set across different training set sizes. This compares the model’s performance when fine-tuned versus trained from scratch over varying training set sizes. The error bars indicate standard deviations, while the central points represent the mean values.

Building on the finding of Zhang et al.,^38^ who demonstrated that fine-tuning significantly enhances model performance in learning aqueous free solvation energies on small experimental data sets, we investigated the effect of training set size on learning tautomer ratios to further illustrate the advantages of fine-tuning over training models from scratch. We created 10 different training sets, ranging from 75 to 300 tautomer pairs, and used them to both fine-tune sPhysNet-Taut and train it from scratch using MMFF94-optimized geometries with 5-fold cross-validation. All models were tested on the 100-tautomers set, and their performance is depicted in Figure 5. Notably, fine-tuning with just 75 tautomer pairs achieved a commendable RMSE of 2.80 kcal/mol, surpassing the performance of models trained from scratch. Furthermore, while the performance of models trained from scratch plateaued as the data set size increased, the fine-tuned models continued to improve with larger data sets. These results affirm that fine-tuning is an effective strategy for training deep learning models on small experimental data sets, offering a promising approach for model development with limited data.

### Ranking Power for Predicting Favorable Tautomers

**Figure 6:**
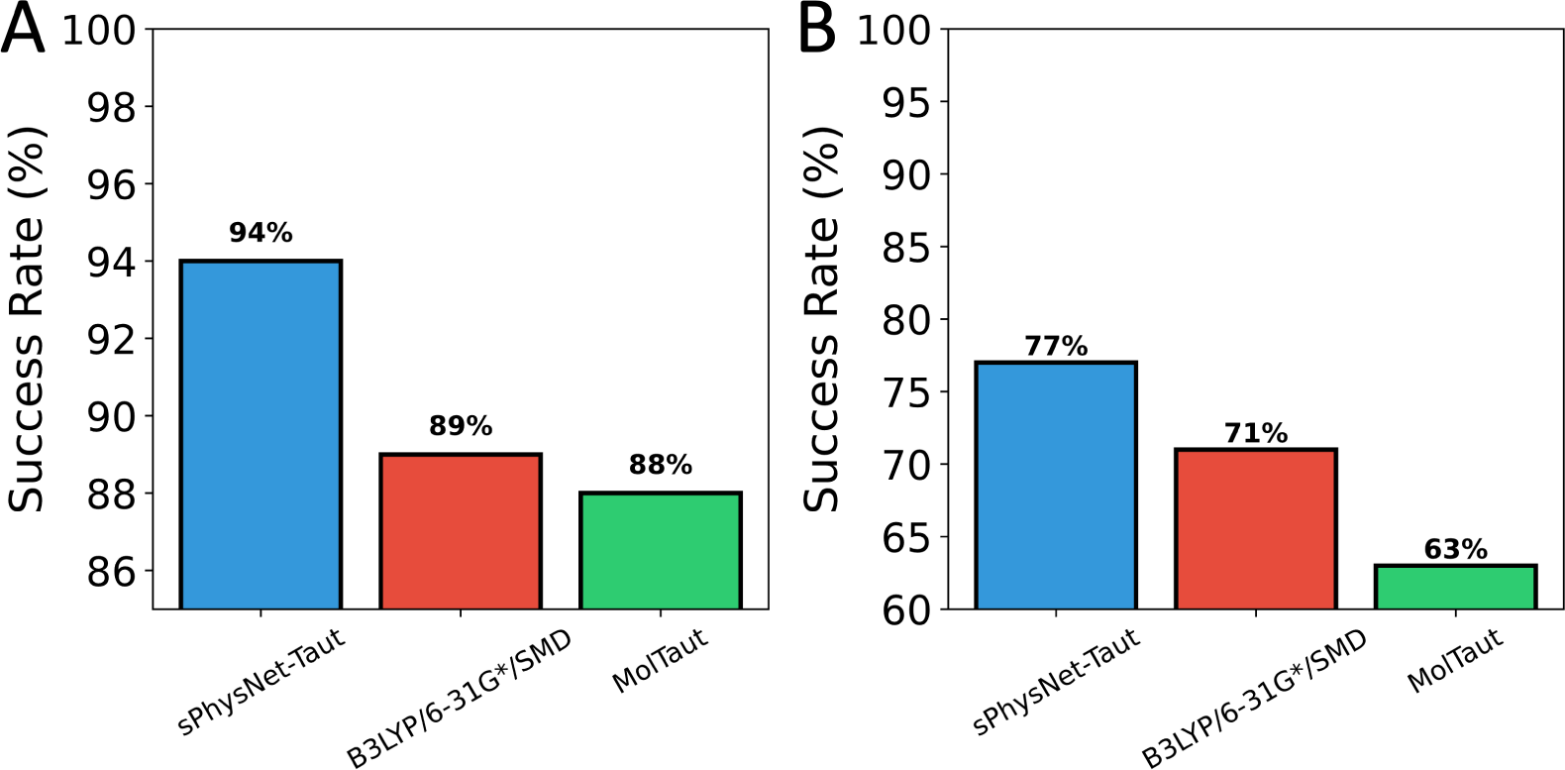
Success rate of ranking tautomer pairs on (A) the 100-tautomers set and (B) the external test set.

To evaluate the ranking ability of our model is also important for its application in drug discovery. We further assessed the ranking ability of sPhysNet-Taut on the two experimental test sets, as presented in Figure 6. On the 100-tautomers set, sPhysNet-Taut accurately ranked 94% of tautomer pairs with MMFF94-optimized geometries, outperforming both the DFT method and MolTaut. To evaluate our model on a larger data set, we selected an external set containing 364 tautomer pairs without logK value from Tautobase, as described in the data set section. On this external test set, sPhysNet-Taut correctly ranked 77% of pairs, again outperforming the DFT method and MolTaut. While the ranking success rate decreased from 94% to 77%, sPhysNet-Taut consistently demonstrated superior performance compared to the other methods.

### Application of sPhysNet-Taut on the PDBbind Database

Tautomerization significantly impacts structure-based and ligand-based screening methods, as different tautomeric states have different pharmacophores, 3D shapes, electrostatic surfaces, and conformations. These variations can lead to different interactions, potentially increasing false positives and computational costs. Generally, the tautomeric state with the lowest energy often dominates, while higher-energy tautomeric states is less abundant in solution and may incur energetic penalties in binding free energy. In this study, we used the PDBbind v2020 refined set^56−58^ to assess the effects of reassigning tautomeric states using sPhysNet-Taut on the hydrogen bonding. To simplify our analysis, we focused solely on neutral molecules in the refined set, identifying 2,086 structures with neutral ligands out of the 5,316 available complex crystal structures available. We excluded crystal structures where the ligands differed from those reported in the original publications. We compared the tautomeric states in the original structures to the lowest-energy tautomeric states predicted by sPhysNet-Taut. We found that 70 original tautomeric states of ligands were more than 2.76 kcal/mol above the predicted lowest-energy tautomeric state, indicating that this state exists in aqueous solution at less than 1%. These findings and the details of the ligands are provided in Table S4. Before analyzing hydrogen bonds, we prepared the structures by removing water molecules, adjusting protein hydrogens, and minimizing all structures with a 0.5 Å RMSD constraint using the Schrödinger protein preparation wizard,^59^ keeping ligand structures unchanged. The number of hydrogen bonds between proteins and ligands was calculated using the Schrödinger Python API.

**Figure 7:**
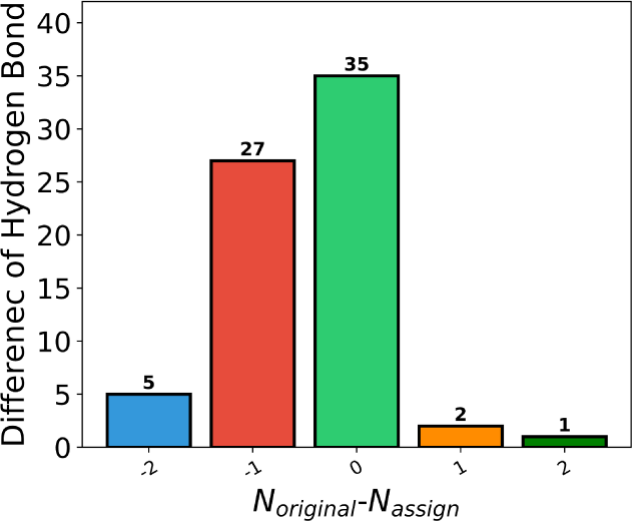
Differences in the number of hydrogen bonds were observed between the original structures in PDBbind and the reassigned tautomer structures generated by sPhysNet-Taut.

Figure 7 illustrates the changes in the number of hydrogen bonds between original structures and reassigned tautomeric states in the PDBbind refined set. Our results show that reassigning to the predicted lowest-energy tautomer increased the number of hydrogen bonds in 32 complex structures, as detailed in Figure S3A. In 35 structures, no changes in hydrogen bonds were observed because the proton shifts occurred at the solvent interface or between nonpolar atoms (Figure S3B), and interactions with bridging water molecules were excluded from the analysis. In three crystal structures, the number of hydrogen bonds decreased following tautomer reassignment. For instance, in the crystal structure of PDB ID 2VK2 (Figure S3C), the predicted conversion of a hydroxyl group to a carbonyl group led to the loss of two hydrogen bonds with ARG17 and GLU13. Additionally, for two other structures (PDB ID: 4G90 and 5OHA), our predictions—though missing one hydrogen bond—are consistent with the original publications but differ from the entries in the PDBbind database, as shown in Figure S3D,E. These findings suggest that reassigning tautomeric states is essential for modeling the protein–ligand interactions.

## Conclusions

Many organic small molecules exist multiple tautomeric states, but typically only one or a few are dominant, which complicates ligand preparation in chemical libraries. Although QM based methods can predict tautomer ratios with high accuracy across various solvents, their substantial computational requirements limit their use in high-throughput applications. To address this challenge, we developed a deep learning model based on a Siamese neural network to predict tautomer ratios in aqueous solution using MMFF94-optimized conformations. We enhanced the pretrained model by fine-tuning it with experimental data to improve its predictions of molecular internal energies and solvent effects. Our pretrained model leverages multitask learning to predict three types of energies: electronic energies in gas and water phases, and transfer energies between these phases, achieving chemical accuracy in these predictions. On the experimental data set, our fine-tuned model achieves state-of-the-art performance, with an RMSE of 1.93 kcal/mol on the test set and 1.16 kcal/mol in the SAMPL2 challenge. It also provides superior ranking power for tautomer pairs on several test sets. This study not only provides a valuable deep learning model for predicting tautomer ratios directly from force field-optimized geometries but also offers a framework for modeling pairwise data. Additionally, we developed a user-friendly tool to estimate all possible tautomeric states using transformation rules and rank them using our sPhysNet-Taut model to predict favorable tautomeric states in aqueous solution. We believe this model will be a handy tool in computational drug discovery by reducing tautomeric conflicts in large chemical libraries, eliminating unstable tautomeric states during virtual screening, and evaluating the effect of substitution on tautomeric equilibrium during lead optimization.

## Data Availability

All data sets and source codes used in this work are available. The Frag20-Taut data set can be accessed at 10.5281/zenodo.13870370, the experimental data used to fine-tune our model is extracted from Tautobase (https://github.com/WahlOya/Tautobase) and the ratio subset and the preference subset) for model training and testing are located at https://github.com/xiaolinpan/sPhysNet-Taut/exp_data. All source codes for data set preparation, model training, and model usage are at https://github.com/xiaolinpan/sPhysNet-Taut. The Web server is located at https://yzhang.hpc.nyu.edu/tautomer.

## Supporting Information Available

Text S1: Detailed description of sPhysNet-MT model; Text S2: Detailed description of the performance for the pretrained model; Text S3: Detailed description of the methods used in the SAMPL2 challenge; Figure S1: Error analysis for different ranges of heavy atom counts; Figure S2: Visualization of molecular structures in the SAMPL2 challenge; Figure S3: Comparative analysis of hydrogen bond interactions between original structures and their reassigned tautomeric states in the PDBbind database; Table S1: Test Performance on the Frag20-Taut calculated test set, which contains 17,844 molecules and 5,844 tautomer pairs; Table S2: Prediction details for each method in the SAMPL2 challenge; Table S3: Data for 70 Ligands from the PDBbind v2020 refined set with tautomeric differences (PDF)
