# Supplementary material for: Fast and Accurate Prediction of Tautomer Ratios in Aqueous Solution via a Siamese Neural Network
Source: J Chem Theory Comput. 2025 Mar 17;21(6):3132–41. doi: 10.1021/acs.jctc.5c00041 (PMC11948319; doi:10.1021/acs.jctc.5c00041)
Supplement: Supplementary file 1 — ct5c00041_si_001.pdf [file ct5c00041_si_001.pdf]

Supporting Information

Fast and Accurate Prediction of Tautomer Ratios in Aqueous Solution via a Siamese Neural Network

Xiaolin Pan[1], Xudong Zhang[1], Song Xia[1], Yingkai Zhang[1,2,3,*]

[1]Department of Chemistry, New York University, New York, 10003, United States
[2]Simons Center for Computational Physical Chemistry at New York University, New York, 10003, United States
[3]NYU-ECNU Center for Computational Chemistry at NYU Shanghai, Shanghai, 200062, China

Email: yingkai.zhang@nyu.edu

Text S1. Description of sPhysNet-MT model

RBF Layer. The electronic energy is highly correlated with molecular conformation, and RBF layers have a great ability to describe the 3D atomic environment. Therefore, we used the RBF layer to encode the 3D information of each conformation. The basic function of the RBF layer is a pairwise distance cutoff function, which is defined as follows:

$$f(r_{ij})\begin{cases}1-6\left(\frac{r_{ij}}{r_{cutoff}}\right)^5+15\left(\frac{r_{ij}}{r_{cutoff}}\right)^4-10\left(\frac{r_{ij}}{r_{cutoff}}\right)^3 & if\ r_{ij}<r_{cutoff}\\ 0 & otherwise\end{cases} \quad (S1)$$

Where r_{ij} represents the Euclidean pairwise distance between the center atom i and the neighbor atom j, r_{cutoff} denotes the cutoff distance, which is set to 10Å.

The geometric information of pairwise atoms is encoded into a vector of $g_k(r_{ij})$ with the distance, which is calculated by K radial basis functions (RBFs) with Gaussian weight. Here is the function form of the RBFs:

$$g_k(r_{ij})=f(r_{ij})\cdot exp\left(-\beta_k\left(exp(-r_{ij})-\mu_k\right)^2\right) \quad (S2)$$

Where $k\in\{1,2,\ldots,64\}$ represents the number of RBFs used to map each distance pair r_{ij} to an embedding vector. β_k and μ_k are learnable parameters in sPhysNet-MT and represent the width and center of RBFs. The μ_k is initialized to a random value between $\exp(-r_{cutoff})$ and 1, and β_k are initialized to the same value, $2K^{-1}(1-exp(-r_{cutoff}))^{-2}$.

Embedding Layer. The embedding layer employs an embedding matrix $e\in R^{94*160}$ to encode the atomic number Z_i of atom i and generate a vector h_i^0. Here, 94 represents the maximum supported atomic number, and 160 donates the bit length of the encoded feature. The embedding matrix e will be updated in the training process. The functional forms of e and h_i^0 are outlined below:

$$e=\begin{bmatrix}x_1^1 & \cdots & x_1^{164}\\ \vdots & \ddots & \vdots\\ x_{94}^1 & \cdots & x_{94}^{164}\end{bmatrix} \quad (S3)$$

$$h_i^0=e[Z_i,:] \quad (S4)$$

Interaction Module. The encoded atomic number relative feature vector h_i^0 and the embedding vectors of the 3D atomic environment $g_k(r_{ij})$ are updated by three interaction modules, as illustrated in **Figure 3B**. The message-passing aggregates the initial embedded vector from neighboring atoms and updates the node embedding of the central atom. The specific details are described as follows:

$$h_i^m=\sigma(W_i\sigma(h_i^m)+b_I)+\sum_{j\neq i}Gg(r_{ij})\circ\sigma\left(W_j\sigma(h_j^m)+b_j\right) \quad (S5)$$

Where $W_i,W_j\in R^{160*160}$, $b_i,b_j\in R^{160}$, and $G\in R^{160\times 94}$ are learnable parameters in the neural network. The activation function $\sigma(x)$ used is the shifted softplus:

$$\sigma(x) = \log(e^x + 1) - \log 2 \tag{S6}$$

Output. The initial vectors $g_k(r_{ij})$ and h_i^0 pass through three interaction modules and a residual module to gain the final atomic embedding vector. This vector is then processed by a linear layer to predict atomic energies, y_i. Ultimately, electronic energies are calculated as the summation of these atomic energies:

$$E = \sum_i^N \left(s_{Z_i} \circ y_i + c_{Z_i}\right) \tag{S7}$$

Where s_{Z_i} and c_{Z_i} are learnable parameters corresponding to different element types, and N represents the total number of atoms in the molecule.

Text S2. Description of the performance for the pre-train model

After training the sPhysNet-MT model on the DFT-calculated dataset following the protocol as described earlier, we evaluated its performance on a test set comprising 17,844 molecules and 5,844 tautomer pairs. The results are detailed in **Table S1**. The performance of sPhysNet-MT in predicting E_{gas}, E_{water}, and ΔE_{trans} surpasses the chemical accuracy threshold of 1.0 kcal/mol, regardless of whether it was trained on geometries optimized by MMFF94 or DFT. Specifically, for the model trained on MMFF94-optimized geometries, the mean absolute errors (MAE) are 0.78 kcal/mol for E_{gas},, 0.75 kcal/mol for E_{water}, and 0.39 kcal/mol for ΔE_{trans}. In contrast, the model trained on DFT-optimized geometries achieves an MAE of 0.36 kcal/mol, 0.36 kcal/mol, and 0.18 kcal/mol for the same properties, respectively. These results highlight that using DFT-optimized geometries for learning single-point energies yields superior performance compared to MMFF94-optimized geometries. Although this outcome is anticipated, obtaining DFT-optimized geometries involves significant computational resources, limiting practical applications. Therefore, using MMFF940optimized structures for learning single-point energies offers a more viable option for real-world applications in drug discovery. Notably, the sPhysNet-MT model also performs well in predicting the relative energies of tautomer pairs. With MMFF94-optimized geometries, the model achieves an MAE of 1.03 kcal/mol and an RMSE of 1.87 kcal/mol. For DFT-optimized geometries, the MAE improves to 0.48 kcal/mol with an RMSE of 0.76 kcal/mol. These results demonstrate that our model effectively learns the relationship between molecular structures and their DFT-calculated electronic energies, accurately capturing the energetic differences between tautomer pairs.

Table S1. Test Performance on the Frag20-Taut calculated test set, which contains 17,844 molecules and 5,844 tautomer pairs

Type	Metric	MMFF94 optimized geometry			DFT optimized geometry		
		E_{gas}	E_{water}	ΔE_{trans}	E_{gas}	E_{water}	ΔE_{trans}
Overall	MAE	0.78	0.75	0.39	0.36	0.36	0.18
	RMSE	1.46	1.43	0.62	0.71	0.74	0.27
		$E_{water}^{t2} - E_{water}^{t1}$					
Tautomer Pairs	MAE	1.03			0.48		
	RMSE	1.87			0.76		

The unit is kcal/mol for all results.

Text S3. Descriptions of the methods on SAMPL2 challenge test set.

Klamt et al.[1] employed BP86/TZVP and COSMO solvent model for geometry optimization, COSMO-BP86/TZVP for free energy in solution, MP2/QZVPP for gas-phase energies, and BP86/TZVP for thermal correction energies, resulting in the root mean square error (RMSE) of 3.35 kcal/mol.

Ribeiro et al.[2] used M06-2X/MG3S to optimize geometry and calculate single-point energy in the gas phase, M06-2X/6-31G(d) to calculate the transfer energies based on 3 different continuum solvent models (SM8, SM8AD, and SMD). The best performance method is M06-2X/MG3S//M06-2X/6-31G(d)/SM8AD with the mean unsigned errors (MUE) of 1.8 kcal/mol.

Kast et al.[3] utilized B3LYP/6-311++G(d,p) and the polarizable continuum solvent model (PCM) to optimize conformation, and then calculated the energy with MP2/aug-cc-pVDZ//ECRISM/PSE-3, it got the best performance with the root mean square error (RMSE) of 2.0 kcal/mol.

Soteras et al.[4] combined MP2/CBS+[CCSD-MP2/6-31+G(d)] calculated gas-phase energies with IEF-MST continuum solvent model calculated transfer energies in water to obtain the RMSD of 3.4 kcal/mol.

Wieder et al.[5] evaluated three quantum chemistry approaches (B3LYP/aug-cc-pVTZ//B3LYP/aug-cc-pVTZ/SMD, B3LYP/aug-cc-pVTZ//B3LYP/6-31G(d)/SMD, and B3LYP/aug-cc-pVTZ/SMD) on Tautobase dataset and achieved the best performance with RMSE of 3.1 kcal/mol using B3LYP/aug-cc-pVTZ//B3LYP/6-31G(d)/SMD.

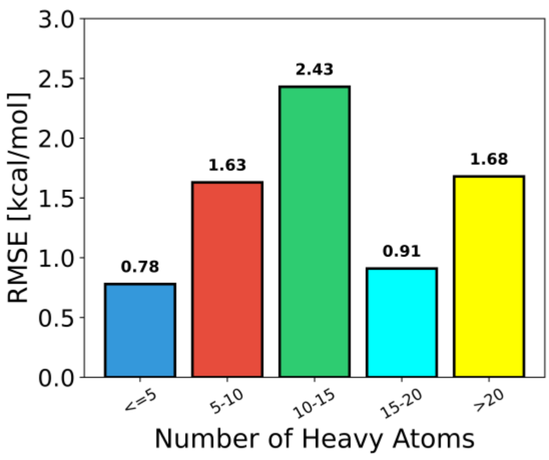

Figure S1. Error analysis across different ranges of heavy atom counts.

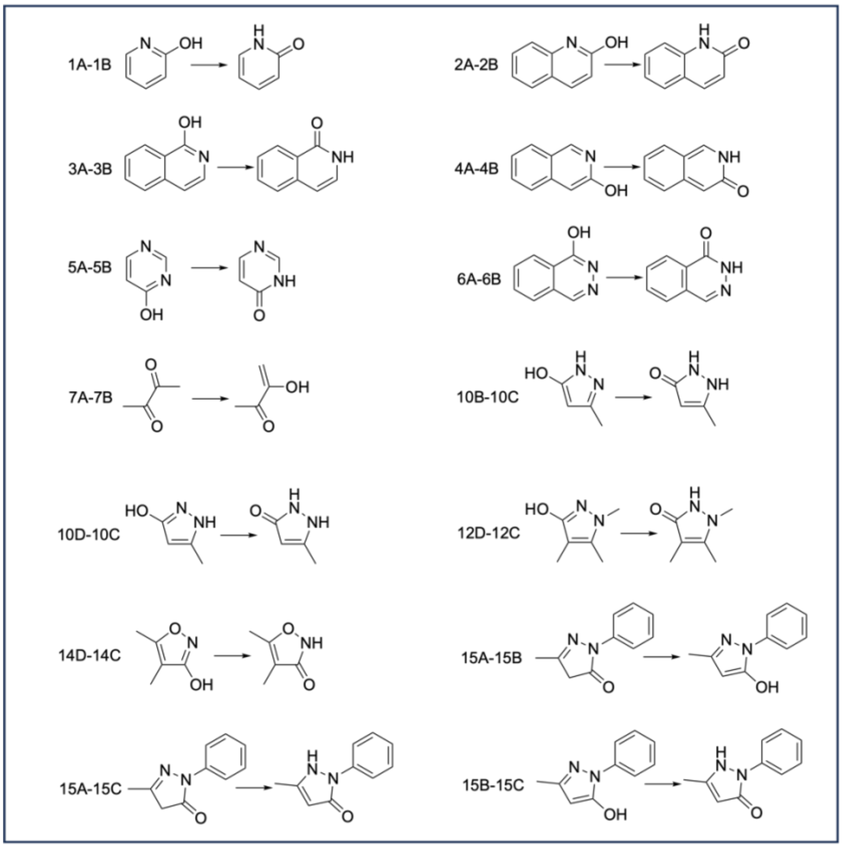

Figure S2. The structures of the SAMPL2 challenge

Table S2. Performance of this work, Wieder et al.'s method, and several submissions of SAMPL2 Challenge

Name	ΔG_{exp}^{t}	sPhysNet-Taut (MMFF94)	MolTaut[a]	Ref1[b]	Ref 2[c]	Ref 3[d]	Ref4[e]	Ref5[f]
1A_1B	-4.8	-5.6	-4.1	-4.7	-4.0	-3.0	-7.7	-4.6
2A_2B	-6.1	-6.5	-6.4	-6.8	-5.7	-5.7	-9.7	-6.3
3A_3B	-7.2	-8.3	-7.4	-8.4	-7.7	-6.7	-11.2	-7.7
4A_4B	-4.8	-2.5	-2.7	-0.4	0.5	0.8	-4.6	0.6
5A_5B	-4.8	-3.8	-2.8	-4.7	-3.9	-4.4	-6.2	-5.6
6A_6B	-9.3	-10.4	-8.4	-11.4	-7.6	-9.7	-11.2	-10.0
**RMSE**		**1.3**	**1.3**	**1.3**	**1.4**	**1.5**	**2.8**	**1.3**
7A_7B	6.6	6.9	8.4	4.9	5.3	6.5	5.1	5.5
10B_10C	-2.9	-2.8	0.3	-5.3	1.7	0.0	-2.8	2.2
10D_10C	-1.2	-1.6	2.5	-1.7	3.8	2.6	-0.6	5.0
12D_12C	-1.8	-1.5	1.2	-2.1	3.3	3.1	-0.8	3.0
14D_14C	0.3	1.0	-0.4	-1.6	1.9	0.8	0.2	4.0
15A_15B	0.9	2.7	6.9	6.1	-3.0	3.6	0.0	0.9
15A_15C	-1.2	-0.0	5.8	0.7	-0.8	2.3	-1.9	1.4
15B_15C	-2.2	-2.7	-1.2	-5.0	1.8	-1.2	-1.9	0.5
**RMSE**		**0.8**	**3.9**	**2.5**	**3.6**	**2.9**	**0.8**	**3.8**
**Total RMSE**		**1.0**	**3.1**	**2.2**	**2.9**	**2.4**	**1.9**	**3.0**

[a]ANI-2x/MolSolv (Pan et al.[6])

[b]B3LYP/aug-cc-pVTZ/B3LYP/6-31G(d)/SMD (Wieder et al.[5])

[c]MP2+vib-CT-BP-TZVP (Klamt et al.[1])

[d]M06-2X/MG3S/M06-2X/6-31G(d)/SM8AD (Ribeiro et al.[2])

[e]MP2/aug-cc-pVDZ/EC-RISM/PSE-3 (Kast et al.[3])

[f]MP2/CBS+[CCSD-MP2/6-31+G(d)](d)/IEF-MST/HF/6-31G (Soteras et al.[4])

Table S3. Detailed Information on the 70 Ligands from the PDBbind v2020 Refined Set with Predicted Tautomeric Differences

pdb id	original	predicted	B3LYP/6-31G*	sPhysNet-Taut
4mhz	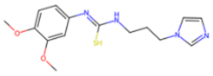	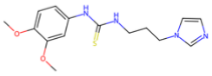	19.40	10.57
4gfo	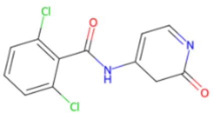	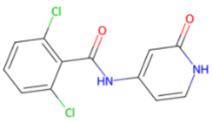	18.57	8.82
4ih7	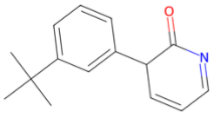	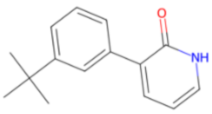	27.75	15.21
1np0	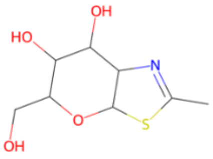	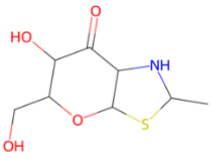	-2.70	3.94
4gfm	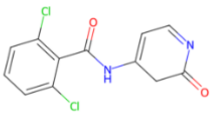	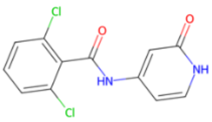	18.57	8.82
5ti0	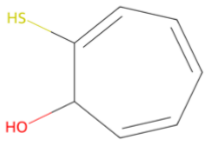	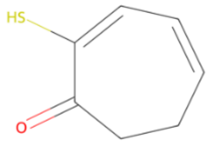	16.29	9.49

2v2q	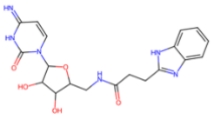	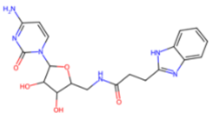	8.77	6.08
5dpx	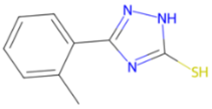	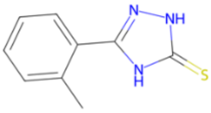	13.95	8.05
6eyb	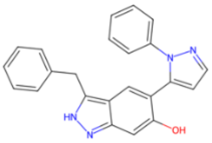	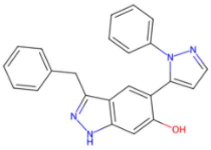	4.09	2.97
4n5d	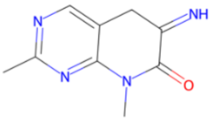	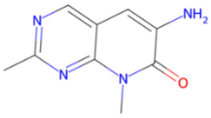	16.01	6.30
4asj	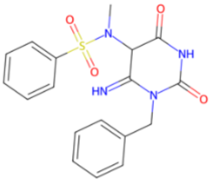	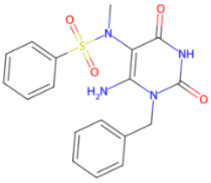	12.33	10.43
3eax	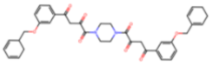	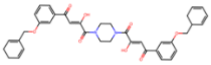	-1.43	8.79
1e3g	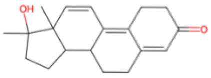	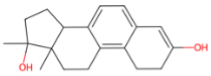	11.39	4.48

1uou	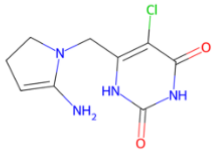	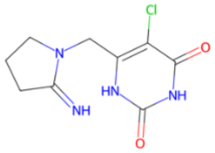	18.67	11.55
3k99	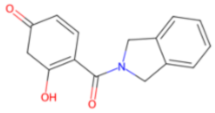	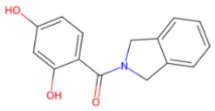	10.21	11.57
6dgr	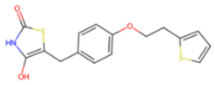	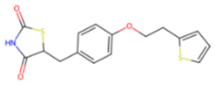	21.23	11.01
4twp	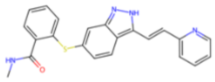	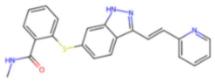	2.06	3.65
1usn	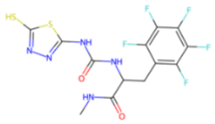	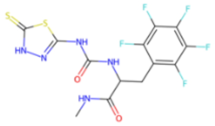	14.06	8.47
3gr2	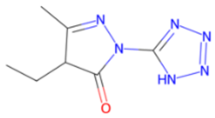	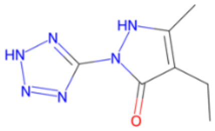	-4.30	1.82
2ygf	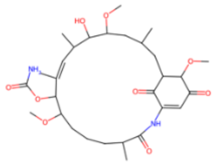	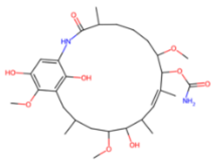	10.19	7.85

5wex	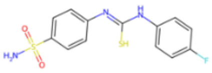	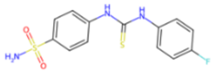	15.17	9.79
3zll	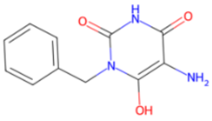	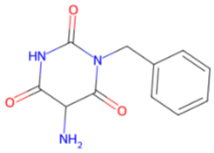	5.01	2.58
4arw	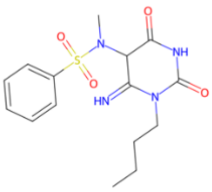	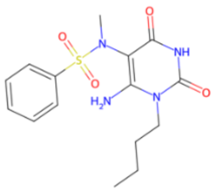	11.68	10.03
5cso	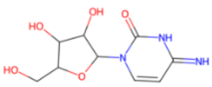	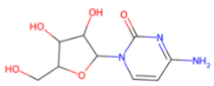	8.80	6.28
4q90	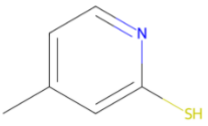	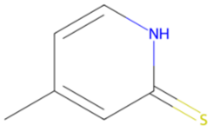	9.50	6.95
4g8y	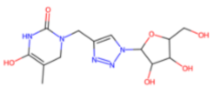	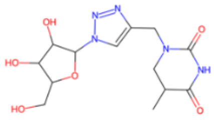	26.27	14.68
6dgl	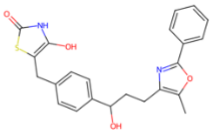	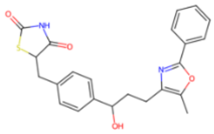	21.47	10.62

4g8v	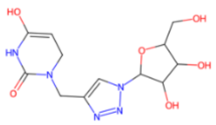	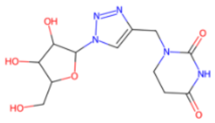	29.48	15.34
3m1k	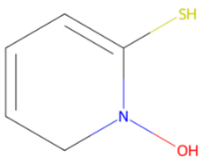	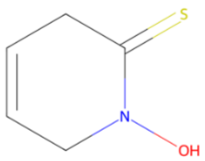	25.15	11.02
2usn	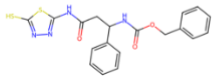	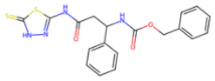	13.86	8.31
3sur, 1hp5, 2epn	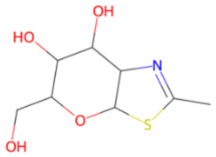	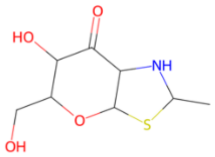	-2.70	3.94
5ndf	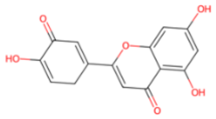	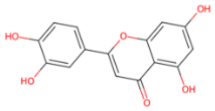	11.67	12.85
5fso	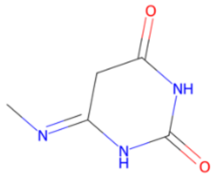	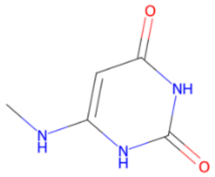	4.42	7.95
6g3q	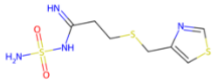	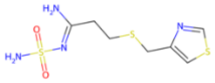	11.50	8.15

2vk2	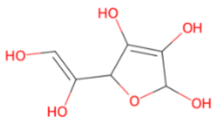	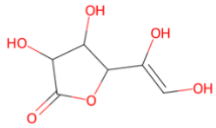	24.97	18.58
1nhz	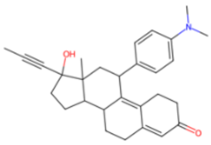	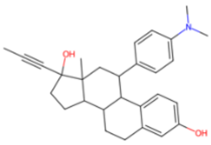	7.42	5.68
2v2v	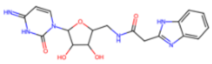	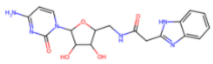	8.64	5.89
4nue	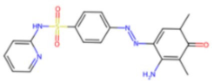	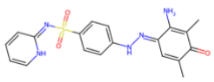	14.73	13.01
5fe9	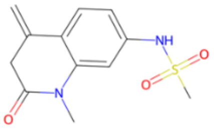	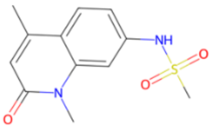	16.40	9.09
4kfq	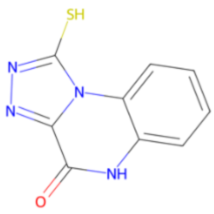	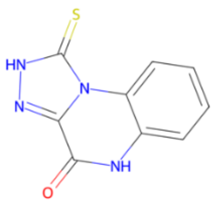	14.30	5.90
3pbb	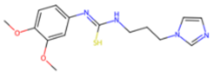	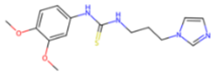	19.40	10.57

4agc	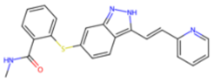	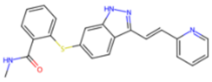	1.97	3.65
5nyh	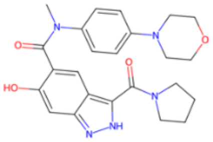	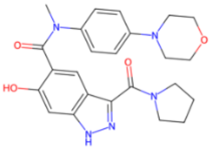	6.77	4.22
4lkq	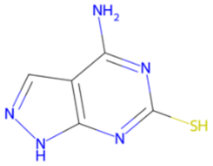	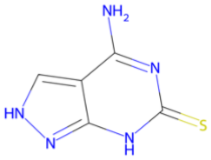	5.08	5.64
6gnp	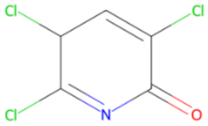	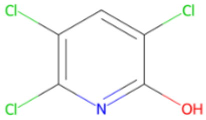	18.34	9.10
4g90	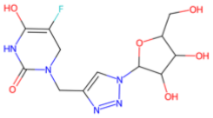	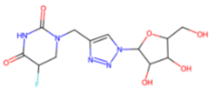	27.84	16.17
6n7d	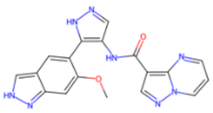	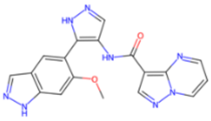	3.56	2.03
5oha	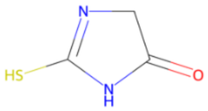	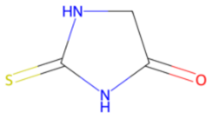	18.16	13.96

5b5f	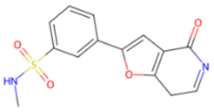	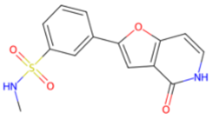	20.56	9.14
4l4v	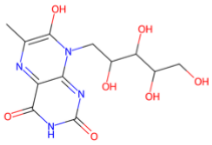	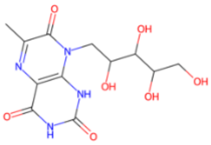	13.73	13.72
4mrw	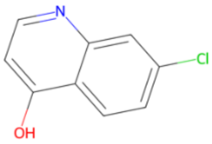	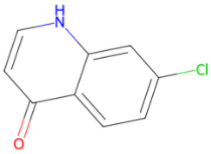	8.47	6.19
4mhy	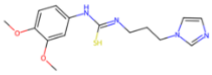	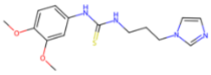	20.99	13.94
3elc	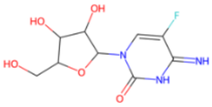	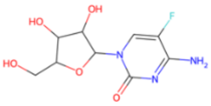	8.20	5.25
2w8y	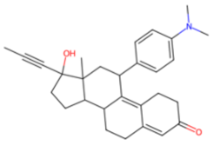	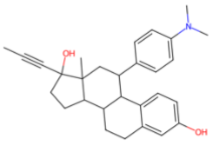	7.43	5.68
6dgq	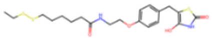	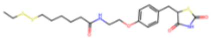	22.37	9.54

2j94	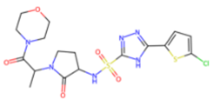	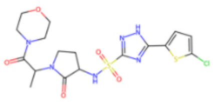	7.85	2.61
2uy3	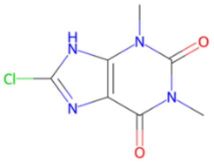	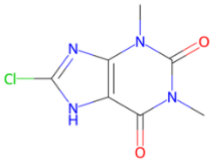	2.51	3.63
2hoc	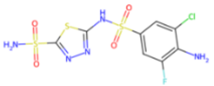	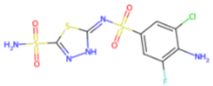	3.45	3.03
4qsu	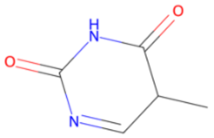	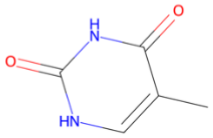	17.38	9.62
4k18	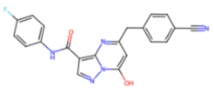	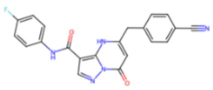	10.54	8.97
4f9u	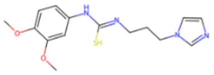	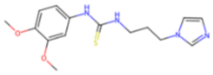	20.99	13.94
2j4g	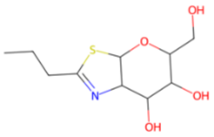	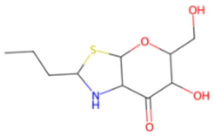	1.65	2.38

3qgy	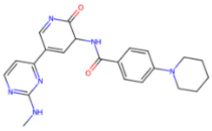	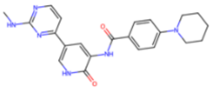	31.42	16.38
5e13	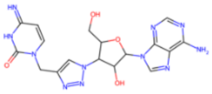	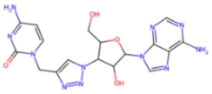	4.72	3.92
4rqk	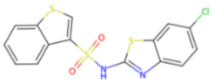	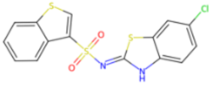	4.05	3.77
3d8w,3dbu	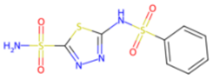	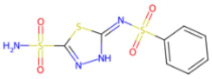	3.45	2.95
1xow	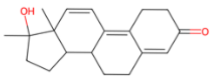	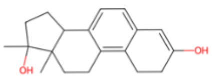	11.39	4.48

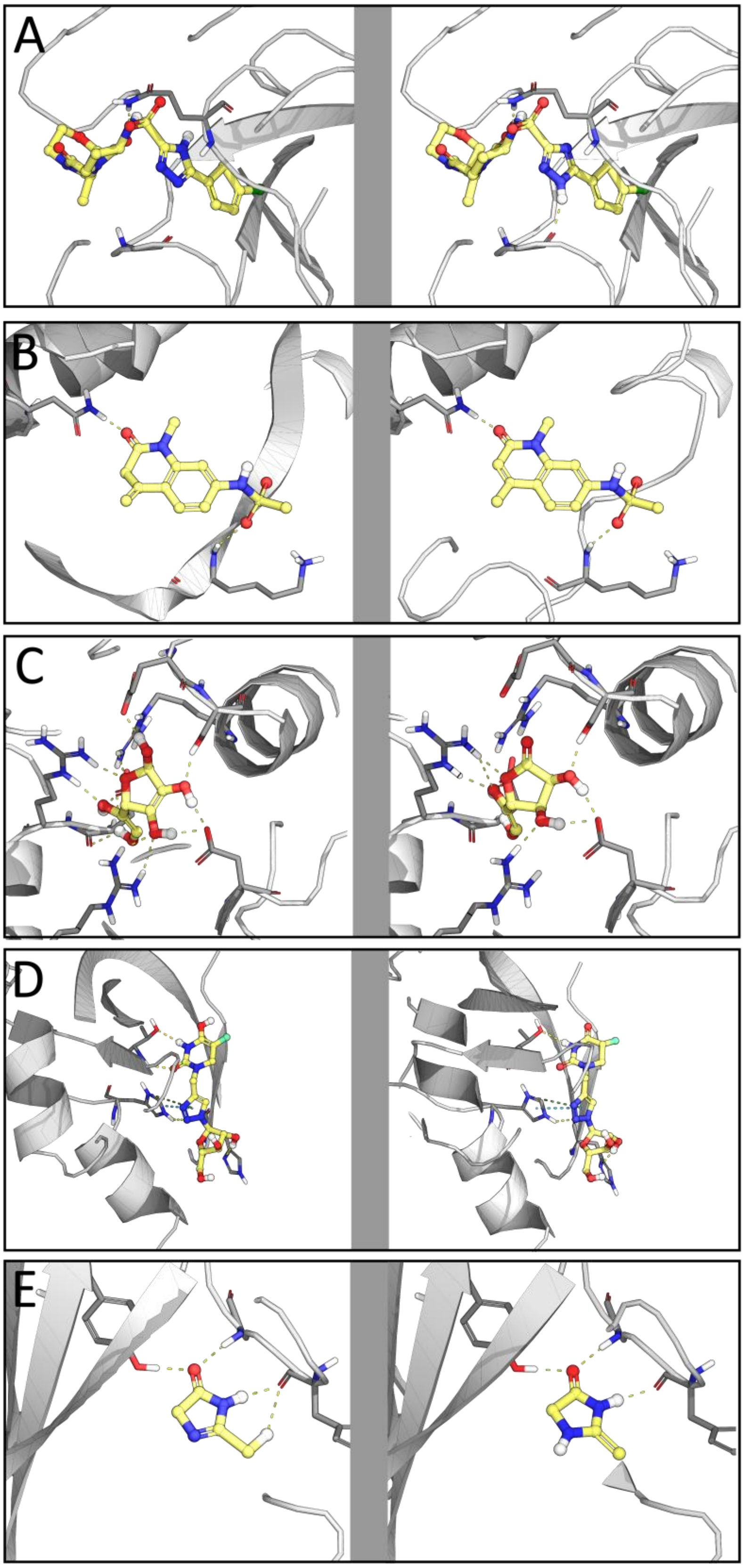

Figure S3. Comparison of the interaction between the original structures in PDBbind (left) and the reassigned tautomer structures (right).

Reference

1. A. Klamt and M. Diedenhofen, *Journal of Computer-Aided Molecular Design*, 2010, **24**, 621-625.
2. R. F. Ribeiro, A. V. Marenich, C. J. Cramer and D. G. Truhlar, *Journal of Computer-Aided Molecular Design*, 2010, **24**, 317-333.
3. S. M. Kast, J. Heil, S. Güssregen and K. F. Schmidt, *Journal of Computer-Aided Molecular Design*, 2010, **24**, 343-353.
4. I. Soteras, M. Orozco and F. J. Luque, *Journal of Computer-Aided Molecular Design*, 2010, **24**, 281-291.
5. M. Wieder, J. Fass and J. D. Chodera, *Chemical Science*, 2021, **12**, 11364-11381.
6. X. Pan, F. Zhao, Y. Zhang, X. Wang, X. Xiao, J. Z. H. Zhang and C. Ji, *Journal of Chemical Information and Modeling*, 2023, **63**, 1833-1840.
